# Knowledge of risk factors for diabetes or cardiovascular disease (CVD) is poor among individuals with risk factors for CVD

**DOI:** 10.1371/journal.pone.0172941

**Published:** 2017-02-28

**Authors:** Monique F. Kilkenny, Libby Dunstan, Doreen Busingye, Tara Purvis, Megan Reyneke, Mary Orgill, Dominique A. Cadilhac

**Affiliations:** 1 Stroke and Ageing Research, School of Clinical Sciences at Monash Health, Department of Medicine, Monash University, Clayton, Victoria, Australia; 2 Stroke Division: Public Health, the Florey Institute of Neurosciences and Mental Health, Heidelberg, Victoria, Australia; 3 Stroke Foundation, Brisbane, Queensland, Australia; 4 Stroke Foundation, Melbourne, Victoria, Australia; 5 The University of Melbourne, Victoria, Australia; Universita degli Studi di Perugia, ITALY

## Abstract

**Background:**

There is limited evidence on whether having pre-existing cardiovascular disease (CVD) or risk factors for CVD such as diabetes, ensures greater knowledge of risk factors important for motivating preventative behaviours. Our objective was to compare knowledge among the Australian public participating in a health check program and their risk status.

**Methods:**

Data from the Stroke Foundation ‘Know your numbers’ program were used. Staff in community pharmacies provided opportunistic health checks (measurement of blood pressure and diabetes risk assessment) among their customers. Participants were categorised: 1) CVD ± risk of CVD: history of stroke, heart disease or kidney disease, and may have risk factors; 2) risk of CVD only: reported having high blood pressure, high cholesterol, diabetes or atrial fibrillation; and 3) CVD risk free (no CVD or risk of CVD). Multivariable logistic regression analyses were performed including adjustment for age and sex.

**Findings:**

Among 4,647 participants, 12% had CVD (55% male, 85% aged 55+ years), 47% were at risk of CVD (40% male, 72% 55+ years) and 41% were CVD risk free (33% male, 27% 55+ years). Participants with CVD (OR: 0.66; 95% CI: 0.55, 0.80) or risk factors for CVD (OR: 0.65; 95% CI: 0.57, 0.73) had poorer knowledge of the risk factors for diabetes/CVD compared to those who were CVD risk free. After adjustment, only participants with risk factors for CVD (OR: 0.80; 95% CI: 0.69, 0.93) had poorer knowledge. Older participants (55+ years) and men had poorer knowledge of diabetes/CVD risk factors and complications of diabetes.

**Conclusions:**

Participants with poorer knowledge of risk factors were older, more often male or were at risk of developing CVD compared with those who were CVD risk free. Health education in these high risk groups should be a priority, as diabetes and CVD are increasing in prevalence throughout the world.

## Introduction

The prevalence of diabetes mellitus (diabetes), and particularly type 2 diabetes, is considered a global epidemic [1]. It is projected to become the leading cause of disease burden for males and the second leading cause for females by 2023 [2]. Diabetes is an independent risk factor for cardiovascular disease (CVD). CVD mainly includes coronary heart disease, heart failure, cardiomyopathy and stroke [3]. About 11.7 million adult Australians (95%) have at least one of the major modifiable risk factors for stroke and heart disease including high blood pressure (BP), diabetes, high cholesterol, smoking, high consumption of alcohol, poor diet, obesity or inadequate physical activity [4]. Furthermore, knowledge of risk factors for CVD and diabetes in the community is poor [5].

The risk factors for diabetes and CVD overlap. Type 2 diabetes results from a combination of genetic and environmental factors. Although there is a strong genetic predisposition to the development of diabetes, the risk is greatly increased when associated with other factors such as high BP, being overweight or obese, physical inactivity and poor diet. It is unclear whether individuals with CVD or risk factors for CVD including diabetes are aware of the risk factors for diabetes/CVD compared to those without CVD or its risk factors. There are limited data on whether individuals with diabetes or who are at high absolute risk of developing diabetes are aware of the complications of diabetes (such as CVD events, amputation, blindness or nerve damage).

Health promotion programs in the community such as the Know your numbers (KYN) program, which involved opportunistic CVD screening assessments in pharmacies, may improve community awareness and knowledge of CVD and associated risk factors such as diabetes [6,7].

Our aim was to compare KYN participants’ knowledge of risk factors for diabetes/CVD and complications associated with diabetes based on their risk status. We hypothesised that knowledge about risk factors or complications would be better in people with pre-existing disease or who were at risk of CVD or diabetes than among those who were risk free. This is because these people with known risk or pre-existing disease should have received some health promotion education if the health system is being effective.

## Methods

### KYN diabetes program

Since 2007, the Stroke Foundation in Australia has undertaken the KYN program which aims to improve public awareness and early detection of individuals at risk of stroke and other CVD [6,7]. The program is designed to facilitate opportunistic CVD and diabetes risk screening assessments in pharmacy and community settings targeting people aged 50+ years. However, subjects aged less than 50 years were still able to participate in the health check program. The features of this program include a standardised BP check, self-reported risk factor checklist, education brochures and doctor referral letter if required. Since 2011 the KYN program has included diabetes risk assessment and educational information on diabetes [8]. The diabetes risk assessment included the participants completing a self-administered screening questionnaire called the Australian Type 2 Diabetes Risk Assessment Tool (AUSDRISK) [9]. A score is calculated from the results of the questionnaire that determines a level of risk for the person developing type 2 diabetes within the next five years. A score of 12 or more means that the participant is at high risk of developing type 2 diabetes within five years or having undiagnosed type 2 diabetes [9]. Participation in KYN provides an important opportunity for members of the community to learn about their CVD and diabetes risk including health-related behaviours (e.g. physical inactivity) or biomedical factors (e.g. high BP) and receive health promotion education [6–8].

### Study design

For this observational study, data from the 2012–2014 KYN program delivered in Australian community pharmacies in the states of Queensland and New South Wales were used. Detailed information was collected from the participant on the day of the health check (called “Participant questionnaire”) from pharmacies who volunteered to collect these data. This questionnaire included information on demographics, self-reported health history, awareness of the KYN health check, diabetes risk assessment and assessment of knowledge of causes of diabetes/CVD and complications of diabetes e.g. stroke or heart attack (Table 1). The questionnaires were developed during the pilot phases of KYN with feedback from the participants [8].

**Table 1:** Example of self-reported medical history and knowledge questions from the Participant Questionnaire collected at the time of the Know your numbers health check.

Your medical history? (Please cross all that apply)		
○ High Blood pressure (hypertension)	○ Diabetes	○ High cholesterol (Dyslipidemia)
○ Previous stroke/ Transient Ischaemic Attack (TIA)	○ Current smoker	○ Ex-smoker
○ Ischaemic heart disease	○ Previous heart disease or heart attack	○ Atrial Fibrillation (AF)
○ Kidney Disease	○ Peripheral vascular Disease	○ None of the above
**What do you think causes diabetes and cardiovascular diseases (e.g. stroke or heart attack)? (Please cross all that apply)**		
○ Being overweight	○ Making unhealthy food choices e.g. not eating enough fruit/vegetables	
○ High blood pressure	○ High cholesterol	○ Smoking
○ Chronic kidney disease	○ Diabetes	○ Family history of CVD or diabetes
○ Being physically inactive (e.g.: less than 30 minutes a day on most days of week)		
○ Drinking too much alcohol (e.g.: more than 2 standard drinks per day)		
○ **Diabetes can lead to serious complications. What are these complications? (Please cross all that apply)**		
○ Heart disease	○ Developing blindness	○ Nerve damage
○ Stroke	○ Amputation	○ Kidney disease
○ Circulation problems	○ Unsure	○

### Covariate measures

#### Risk factor measurements

The AUSDRISK was used as part of the assessment of risk of diabetes within the KYN program [8]. The AUSDRISK score and the BP reading were recorded on the participant questionnaire on the day of the health check. A participant was classified as **High Risk** if they recorded a high BP reading (≥140/90 mmHg) or a high AUSDRISK score of 12 or more.

**Socioeconomic status (SES)** was defined using the Index of Relative Socioeconomic Disadvantage (IRSD) [10]. Pharmacy address was used as a surrogate for a participant’s place of residence. These were matched to local government area (LGA) and then matched to the IRSD and corresponding quintiles were derived. Quintile 1 (IRSD 1) refers to area of most socioeconomic disadvantage and Quintile 5 (IRSD 5) refers to area of least socioeconomic disadvantage. ***High SES***: includes quintiles 4 and 5 of the Index of Relative Socioeconomic Disadvantage.

#### Knowledge assessment: Risk factors for diabetes/CVD

Participants were asked to select from a pre-defined list of ten risk factors for diabetes and CVD (Table 1). Knowledge scores were calculated for each participant based on the number of options selected. Participants received one point for each of the ten response options. Knowledge scores ranged from 0 to 10.

#### Knowledge assessment: Complications of diabetes

Participants were asked to select the complications of diabetes from a pre-defined list (Table 1). Knowledge scores were calculated for each participant based on the number of options selected as complications of diabetes. Participants received one point for each of the seven complications of diabetes correctly selected. Knowledge scores ranged from 0 to 7.

### Definition of subject groups

- **CVD risk status**: Participants were categorised into the following CVD risk groups based on their self-reported medical history (Table 1): 1) ***CVD*** *±* ***risk of CVD*** included stroke/transient ischaemic attack (TIA), heart disease/heart attack, peripheral vascular disease, ischaemic heart disease, or kidney disease. They may have also reported risk factors including high BP, high cholesterol, diabetes, or atrial fibrillation; 2) ***Risk of CVD*** only included high BP, high cholesterol, diabetes or atrial fibrillation; 3) ***CVD risk free*** if the participant did not report any of these options.
- **Diabetes risk status**: Participants were categorised into the following diabetes risk groups based on their self-reported medical history (Table 1) or their AUSDRISK score. 1) ***Diabetes*** included self-reported diagnosis of diabetes; 2) ***Risk of diabetes*** if the participant did not have a self-reported diagnosis of diabetes but recorded a high AUSDRISK score of 12 or more; 3) ***Diabetes risk free*** if the participant did not have a self-reported diagnosis of diabetes and recorded an AUSDRISK scored less than 12.

### Analyses

Chi-square test for categorical variables and Kolmogorov Smirnov test for continuous data were used to compare participant characteristics and knowledge of risk factors and complications. To determine whether history of CVD or diabetes risk was associated with greater knowledge, univariable and multivariable logistic regression analyses were performed using the knowledge score (lesser knowledge group coded as zero and greater knowledge group coded as one) as a dependent variable. The lesser knowledge group were those with a knowledge score below the median score while the greater knowledge group had a knowledge score at or above the median score. The multivariable models were adjusted for risk status, age, sex, socioeconomic status and high risk (based on results of health check). Level of significance was p<0.05. All analyses were performed using Intercool STATA 12.1 for Windows software (Stata Corp PL, 2013).

### Ethics

Ethics approval for the evaluation was provided by the Monash University Human Research Ethics Committee (HREC CF12/1103–2012000528). Written informed consent was not obtained from the participant to collect detailed information on the “Participant questionnaire”. However, consent was implied, as evidenced that they completed the form. Information was collected anonymously from participants and was de-identified in these analyses.

## Results

Questionnaire data were completed by 4,647 participants from pharmacies in Queensland and New South Wales (Australia) participating in the KYN program from 2012 to 2014. More than half of the participants (55%) were aged over 54 years and 39% were male. Following the KYN health check, 2,318 participants (50%) were assessed to be at high risk. One third of participants (37%) recorded a high BP and 45% were assessed as having a high 5-year risk of developing diabetes (AUSDRISK score 12+). Of the participants who had CVD, one third reported history of stroke/TIA; 23% ischaemic heart disease; 20% kidney disease; 15% peripheral vascular disease and 49% heart disease/heart attack. The participants who had CVD also reported the following risk factors: 76% had high BP; 47% high cholesterol; 30% diabetes and 14% atrial fibrillation. Of the participants who were categorised as at risk of CVD only: 83% had high BP; 37% high cholesterol; 22% diabetes and 5% atrial fibrillation.

### CVD risk status

Univariable analyses of the participant characteristics according to CVD status are provided in Table 2. Participants attending a pharmacy in an area of relative socioeconomic advantage (high SES) were more often CVD risk free. Compared with participants who were CVD risk free, participants with pre-existing or risk of CVD were more often older (55 years or more), male and had recorded a high BP reading or high AUDRISK score on the day of the health check.

**Table 2:** Characteristics of participants by CVD risk status.

Characteristics	CVD^1^ (N = 552) n (%)	Risk of CVD^2^ (N = 2,185) n (%)	CVD risk free^3^ (N = 1,910) n (%)	p-value CVD vs CVD Risk free	p-value Risk of CVD vs CVD Risk free
**Age group (years)**				<0.001	<0.001
<34 years	7 (1)	57 (3)	576 (31)		
35–44	18 (3)	160 (7)	343 (18)		
45–54	57 (10)	388 (18)	436 (23)		
55–64	107 (20)	640 (30)	295 (16)		
65–74	195 (36)	567 (26)	151 (8)		
75+	162 (30)	356 (16)	66 (4)		
Age 55+ years	464 (85)	1,563 (72)	512 (27)	<0.001	<0.001
**Patient characteristics**					
Male	293 (55)	849 (40)	608 (33)	<0.001	<0.001
Australian	400 (72)	1,470 (67)	1,308 (68)	0.07	0.41
Aboriginal/Torres Strait Islander	15 (3)	37 (2)	38 (2)	0.30	0.48
**Socioeconomic status (SES)**				0.15	0.002
IRSD 1	66 (12)	359 (17)	233 (13)		
IRSD 2	112 (21)	369 (17)	300 (16)		
IRSD 3	135 (25)	530 (25)	478 (26)		
IRSD 4	142 (26)	539 (25)	511 (27)		
IRSD 5	86 (16)	353 (16)	340 (18)		
High SES (IRSD 4/5)	228 (42)	892 (41)	851 (45)	0.14	0.007
**Results of KYN check**					
High 5-year risk* diabetes	218 (81)	816 (63)	355 (23)	<0.001	<0.001
High BP reading (≥140/90 mmHg)	228 (46)	990 (49)	373 (21)	<0.001	<0.001
High BP/High 5-year risk diabetes*	346 (63)	1,374 (63)	598 (31)	<0.001	<0.001

CVD: Cardiovascular disease

^1^CVD ± risk of CVD: heart disease, heart attack, stroke/transient ischaemic attack, ischaemic heart disease, kidney disease, and may have high blood pressure, high cholesterol, diabetes, atrial fibrillation

^2^Risk of CVD only: high blood pressure, high cholesterol, diabetes atrial fibrillation

^3^CVD risk free: no CVD or risk of CVD; KYN: Know your numbers program

*based on AUSDRISK score 12+; IRSD: Index of Relative Socioeconomic Disadvantage.

Univariable analyses of knowledge according to CVD status on the same day of the health check are provided in Table 3. Compared with participants who were CVD risk free, participants with CVD or risk of CVD more often had poorer knowledge of risk factors for diabetes/CVD such as being physically inactive, or having diabetes. The median knowledge score was 6 (Q1: 3, Q3: 9 [Q1: 25^th^ percentile, Q3: 75^th^ percentile]). To assess factors associated with knowledge of risk factors, participants were divided into two groups: the group with the lesser knowledge (knowledge median score <6) and the group with greater knowledge (knowledge median score 6+) in multivariable analyses (Table 4).

**Table 3:** Univariable analysis of knowledge of risk factors for CVD by CVD risk status.

Risk factors for diabetes/CVD	CVD^1^ (N = 552) n (%)	Risk of CVD^2^ (N = 2,185) n (%)	CVD Risk free^3^ (N = 1,910)n (%)	p-value CVD vs CVD Risk free	p-value Risk of CVD vs CVD Risk free
Being physically inactive	322 (59)	1,263 (58)	1,258 (67)	<0.001	<0.001
Unhealthy foods	345 (63)	1,392 (64)	1,328 (70)	0.001	<0.001
Being overweight	444 (81)	1,840 (85)	1,630 (86)	0.001	0.20
Kidney disease	167 (30)	551 (25)	601 (32)	0.51	<0.001
High blood pressure	397 (72)	1,573 (73)	1,347 (71)	0.70	0.38
Diabetes	228 (41)	911 (42)	964 (51)	<0.001	<0.001
Drinking too much alcohol	279 (51)	1,022 (47)	1,102 (58)	0.001	<0.001
High cholesterol	340 (62)	1,280 (59)	1,233 (65)	0.13	<0.001
Smoking	328 (60)	1,251 (58)	1,287 (68)	<0.001	<0.001
Family history	345 (63)	1,339 (62)	1,325 (70)	0.001	<0.001
***Knowledge score***				0.001	<0.001
0	32 (6)	111 (5)	96 (5)		
1–3	123 (22)	499 (23)	313 (17)		
4–7	191 (35)	800 (37)	614 (33)		
8–10	203 (37)	755 (35)	863 (46)		
Median score (Q1, Q3)	6 (3, 9)	6 (3, 9)	7 (4, 9)	<0.001	<0.001

CVD: Cardiovascular disease

^1^CVD ± risk of CVD: heart disease, heart attack, stroke/transient ischaemic attack, ischaemic heart disease, kidney disease and may have high blood pressure, high cholesterol, diabetes atrial fibrillation

^2^Risk of CVD only: high blood pressure, high cholesterol, diabetes atrial fibrillation

^3^CVD risk free: no CVD or risk of CVD. Q1: 25^th^ percentile; Q3: 75^th^ percentile.

**Table 4:** Multivariable analysis of factors associated with knowledge of risk factors for diabetes/CVD.

Median knowledge score of 6+	Univariable analysis OR (95% CI)	p-value	Multivariable analysis OR (95% CI)	p-value
Age (55+ years)	**0.59 (0.52, 0.66)**	<0.001	**0.69 (0.60, 0.79)**	<0.001
Male	**0.75 (0.66, 0.84)**	<0.001	**0.80 (0.70, 0.91)**	0.001
High BP/High 5-year risk diabetes* (at health check)	**0.64 (0.53, 0.76)**	<0.001	**0.79 (0.70, 0.90)**	<0.001
***CVD risk status***				
CVD risk free^3^	1.0 (reference group)		1.0 (reference group)	
***Risk of CVD***^***2***^	**0.65 (0.57, 0.73)**	<0.001	**0.80 (0.69, 0.93)**	0.003
***CVD***^***1***^	**0.66 (0.55, 0.80)**	<0.001	0.87 (0.70, 1.08)	0.20
High SES^4^	1.07 (0.95, 1.21)	0.24	1.07 (0.95, 1.21)	0.28

CVD: Cardiovascular disease

^1^ CVD ± CVD risk: heart disease, heart attack, stroke/transient ischaemic attack, ischaemic heart disease, kidney disease and may have high blood pressure, high cholesterol, diabetes, atrial fibrillation

^2^Risk of CVD only: high blood pressure, high cholesterol, diabetes atrial fibrillation

^3^CVD risk free: no CVD or risk of CVD

*based on AUSDRISK score 12+

^4^High SES: quintile 4 and 5 of the Index of Relative Socioeconomic Disadvantage; Multivariable model adjusted for age, sex, high BP/high 5-year risk diabetes and CVD risk status.

Multivariable analyses of knowledge of risk factors of diabetes/CVD according to CVD status are provided in Table 4. Interestingly, having CVD or risk factors for CVD was associated with poorer knowledge of the risk factors for diabetes/CVD. However, on adjustment for age, sex, high risk (based on health check) and socioeconomic status, this association remained in individuals with risk factors for CVD only.

### Diabetes risk status

Compared with participants who were diabetes risk free, participants with risk of diabetes were more likely to have poor knowledge of complications of diabetes such as stroke or circulation problems. The median knowledge score was 4 (Q1: 2, Q3: 6). Participants were divided into two groups, with those with poor knowledge having a score less than four (the median knowledge score), and the group with greater knowledge having a median knowledge score of 4 or more (Table 5).

**Table 5:** Univariable analysis of knowledge of complications of diabetes by diabetes risk status.

Complications of diabetes	Diabetes^1^ (N = 634) n (%)	Risk of diabetes^2^ (N = 1,389) n (%)	Diabetes risk free^3^ (N = 1,702) n (%)	p-value Diabetes vs Diabetes risk free	p-value Risk of diabetes vs Diabetes risk free
Kidney disease	315 (50)	636 (46)	923 (54)	0.06	<0.001
Nerve damage	294 (47)	573 (41)	819 (48)	0.48	<0.001
Amputation	188 (30)	432 (31)	526 (31)	0.58	0.88
Developing blindness	392 (62)	819 (59)	1,082 (64)	0.47	0.01
Circulation problems	401 (63)	864 (62)	1,180 (69)	0.006	<0.001
Stroke	398 (63)	814 (59)	1,095 (64)	0.52	0.001
Heart disease	445 (70)	894 (65)	1,150 (68)	0.20	0.07
Unsure	48 (8)	92 (7)	114 (7)	0.45	0.94
***Knowledge score***				0.28	<0.001
0	52 (8)	127 (9)	152 (9)		
1–3	229 (36)	557 (40)	557 (33)		
4–7	351 (56)	701 (51)	991 (58)		
Median score (Q1, Q3)	4 (2, 6)	4 (2, 6)	4 (2, 6)	0.21	<0.001

^1^Diabetes: reported in medical history

^2^Risk of diabetes: AUSDRISK score recorded 12+

^3^Diabetes risk free: no diabetes, or AUSDRISK score recorded <12; Q1: 25^th^ percentile; Q3: 75^th^ percentile.

In contrast, having diabetes was not independently associated with good knowledge of the complications of diabetes (Table 6) compared to those who were diabetes risk free. Individuals with risk factors for diabetes were associated with having poorer knowledge of the complications of diabetes compared to those who were diabetes risk free. However, this association did not remain after adjustment for age, sex, socioeconomic position and diabetes risk status. In all analyses, being older (aged 55 years or more) and male were consistently associated with poorer knowledge of the complications of diabetes.

**Table 6:** Multivariable analysis of factors associated with greater knowledge of complications of diabetes.

Median knowledge score of 4+	Univariable analysis OR (95% CI)	p-value	Multivariable analysis OR (95% CI)	p-value
Age (55+ years)	**0.66 (0.58, 0.74)**	**<0.001**	**0.72 (0.62, 0.83)**	<0.001
Male	**0.75 (0.66, 0.84)**	**<0.001**	**0.77 (0.67, 0.89)**	<0.001
Diabetes status				
Diabetes risk free^1^	1.0 (reference group)		1.0 (reference group)	
***Risk of diabetes***^***2***^	**0.73 (0.64, 0.85)**	<0.001	0.88 (0.75, 1.03)	0.11
***Diabetes***^***3***^	0.89 (0.74, 1.07)	0.23	1.14 (0.93, 1.40)	0.21
High SES^4^	0.92 (0.82, 1.04)	0.17	0.98 (0.86, 1.12)	0.80

^1^Diabetes risk free: no diabetes, or AUSDRISK score recorded <12

^2^Risk of diabetes: AUSDRISK score recorded 12+

^3^Diabetes: reported in medical history

^4^High SES: quintile 4 and 5 of the Index of Relative Socioeconomic Disadvantage; Multivariable model adjusted for age, sex, socioeconomic position and diabetes risk status.

## Discussion

Evidence about individuals’ knowledge of risk factors associated with diabetes/CVD and complications of diabetes is rare. This study contributes new knowledge for developed countries on the association between knowledge of risk factors for diabetes/CVD and complications of diabetes in individuals with pre-existing disease or who are at risk, and those who were risk free. The findings provide evidence that knowledge of risk factors for diabetes/CVD was poorer in participants who were at risk of CVD who were more often male and over 55 years. The factors associated with poor knowledge of risk factors of CVD/diabetes were older age, being male and those participants who recorded a high BP or risk of diabetes (at the health check).

Our study contrasts with a United States study of 4,193 healthy people in which knowledge of CVD risk factors was associated with presence of risk factors [11]. However, this study was conducted in young adults (mean age 30 years), compared to our study where half of the participants were aged 55 years or older. Our findings were similar in univariable analyses to those of a study from China in which knowledge of risk factors for stroke was reported to be poorer in patients with previous stroke or TIA [12]. Although risk of diabetes was not independently associated with knowledge of complications, this does not negate the fact that individuals with pre-existing diabetes were more likely to mention the following complications of diabetes: kidney disease, nerve damage, blindness, circulation problems and stroke. This further highlights the need for public health awareness programs such as KYN, aimed at educating the public about CVD risks and complications, particularly those related to diabetes.

Previous research shows that older age, lower educational status and limited English language are associated with poor knowledge of diabetes/CVD risk factors or complications [13]. In one study, it was reported that age had a negative association with knowledge of risk factors for diabetes in a study conducted in Saudi Arabia [14]. However, in a small Australian study of 181 patients with type 2 diabetes, there was no association found between older age and knowledge of diabetes [13]. In our study, educational status and English fluency were not collected as part of our participant questionnaire, so we were unable to adjust for these factors in our regression models. Nevertheless, there is substantial evidence from other studies that show lower socioeconomic position, measured by level of education, to be associated with poorer knowledge of risk factors [11,13].

Our findings highlight poor knowledge of risk factors for diabetes and CVD in a high risk group. Given that better knowledge of diabetes is associated with ability to perform self-care activities (e.g. proper diet, regular exercise and blood glucose monitoring) [15], and better medication adherence and glycaemic control [16], this underscores the need for patient and community education programs for diabetes. Moreover, educational interventions for diabetes have been shown to improve patients’ knowledge about diabetes, clinical, behavioural, and patient-centred outcomes and complications associated with diabetes [17–19]. Perhaps, as we have shown previously, volunteer-led community interventions such as the NSF StrokeSafe Ambassador Program could be adopted to improve knowledge and awareness of diabetes and CVD risk factors and complications of diabetes in high risk groups as well as in communities [20]. The Ambassador program included presentations by volunteers trained to be ‘ambassadors’ to spread standard information about stroke to the public on stroke risks and warning signs.

The potential limitations of the KYN data have been noted in previous publications as related to the KYN program [6–8]. In brief, the group of participants being tested was a convenience sample and may not be representative of the general population. The questionnaires used were pilot tested and refined based on feedback from participants, KYN staff and the working group to ensure face validity including readability and completeness. Other forms of reliability and validity were not undertaken. We also may have overestimated levels of knowledge by using close-ended questions in which specific risk factors were listed as opposed to using open-ended questions in the participant questionnaire. The strengths of our study include the large sample of pharmacies and community-based participants.

Increasing awareness of CVD and diabetes in the population is important, given the high proportion of participants with pre-existing CVD/diabetes or at risk of CVD/diabetes who were unaware of the risk factors and complications of diabetes. There is a need for further education of people who attend health checks who record a high BP or risk of diabetes, as these people have inadequate knowledge of risk factors for CVD/diabetes. Diabetes Australia have developed education programs related to diabetes and complications [13]. These education programs could be modified to target older individuals and men in these high risk groups to improve knowledge and awareness of diabetes and CVD.

In summary, the findings of this study suggest that individuals who are at risk of CVD, male or over 55 years have poorer knowledge of risk factors for CVD/diabetes. Targeting these groups with tailored educational information may help improve knowledge of risk factors and their understanding of the complications of diabetes.
